# Hospital and Institutional News

**Published:** 1920-01-24

**Authors:** 


### HOSPITAL AND INSTITUTIONAL NEWS.

### THE POTTERIES' SPLENDID AWAKENING.

We are pleased to see from the Annual Report of the North Staffordshire Infirmary, Stoke-on-Trent, being that of the Committee to the Governors, and from the speeches made at the Governors' annual meeting, that the above-mentioned hospital is still forging ahead and continuing to build upon the sure foundations laid in 1911 when new rules and regulations were adopted and up-to-date buildings were erected. That the hospital has become firmly established, in the hearts of the general public of the district, is shown by the further increase in annual subscriptions, especially from the industrial workers. In 1909-10 the ordinary annual income was about £11,000; last year it was £29,000. Workmen's subscriptions and Hospital Saturday collections in 1909-10 amounted approximately to £6,000; last year more than £18,000 was received from these sources. Legacies and donations (over £6,000) showed a marked increase, and the receipt of £9,355 for "Public Services" shows that this hospital was able to render substantial service to our wounded and discharged soldiers and sailors. The number of beds has been increased by 35 to 280, but the waiting-list still exists and should be abolished. Birmingham's lead, explained in another column, might show the authorities a way out. The arrangements for the nursing staff urgently demand immediate reform; the salaries paid are quite inadequate, and the hours of work excessive, and should be changed forthwith by a hospital which has accomplished so much in the way of reform and improvement since 1910. North Staffordshire has a grand opportunity to wake up the 90 per cent. of the residents, most of whom have given nothing so far to the support of the voluntary hospitals. We have now 250 centres where the waking-up process and the organisation of means to reach the whole of the people resident in the districts served by great hospitals is in an active state of incubation. North Staffordshire has set its heart upon providing a Peace Memorial for the Potteries, and we hold that none can be suggested likely to prove more enduring in the hearts of the people of the Potteries, than the provision and extension of their hospital and its establishments, and the placing of them once and for all upon a sound financial basis. Then this memorial of voluntary enterprise and self-sacrifice would indeed be magnificent. The annual meeting showed the vitality of the hospital movement in the Potteries. We rejoice in the re-election of Mr. J. Taylor Howson as President. He has, we know, the co-operation of the people of the district, who, under the guidance of the Mayors of Stoke-on-Trent and Newcastle-under-Lyme, will no doubt prove once more, by their keen enthusiasm for the welfare of the hospital and its best interest, what the potters can do, when they put their shoulders to the wheel, in providing an institution which will win the admiration of the kingdom, as their ware has been the admiration of the world.

### RONTGEN RAY EXHIBITION

No more striking illustration of the rapidity of modern scientific advance and the enormous economic value thereof could be given than the exhibition of prints taken by the Röntgen rays and shown at the rooms of the Royal Photographic Society. Side by side one sees the first *x*-ray photograph of the human hand, taken in London in 1896, requiring an exposure of something like 20 minutes, and a similar radiograph obtained in 1919 in one hundredth part of a second. The medical and surgical application of the rays are well known to the majority of our readers, but, even so, a visit to this exhibit will have demonstrated many things that are new to them. Also the, to us, less familiar applications were of absorbing interest. Among them may be mentioned the detection of flaws in the welding of metals, a process of vital importance in this era of mechanical power; the perfecting of finger-print technique; and even that most remarkable application of Dr. Heilbroun of Amsterdam, who has subjected oil painting to the rays and so detected the presence of later additions superimposing the work of the original artist. The possible uses to which this agency may be turned are probably even yet far from exhausted, and, be he doctor or pure scientist, the visitor is well rewarded by the interest and promptings this exhibition provides. The exhibition will remain open until February 7.

### SEA-LIFE AND TUBERCULOSIS.

It may seem strange to learn that seamen need a sanatorium chiefly because sailors suffer severely from consumption. The sea is supposed to be the symbol of open-air life? Can one then have too much even of the best thing: can even the open air be overdone? That is not the experience of sanatorium superintendents, though it is true these now incline perhaps to mitigate the extreme discomforts of the open-air treatment in winter by giving more protection to the beds than was sometimes the case when in an extravagance of air-worship they were draped by a coverlet of snow The truth seems to be not only that there is a difference between an open-air life and a life of exposure, but that the odd thing about a ship is that it is usually the stuffiest structure in the world. Who that has ever been on board ship has not hesitated between the exhausted air of saloon or cabin and the icy rigours of the deck? If this is true from the point of view of the passenger must not the contrast be even more disagreeably acute to the seaman? Sea life oscillates between exposure and exhausted air: hence the sailor becomes an unhappy hunting-ground for the tuberculosis bacillus.

### MEMORIAL TO LORD LISTER

A PUBLIC appeal has been made by the University of Edinburgh and the Royal College of Physicians and Surgeons, Edinburgh, with a view to erecting a research institute as a memorial to Lord Lister in Edinburgh. The sum required is £250,000, and towards this the University and the Royal College have already contributed sums amounting to £25,000.

### MESOPOTAMIAN DEMOBILISATION.

SLOWLY indeed does medical demobilisation appear to be taking place from the Mesopotamia area, although at most of our London hospitals little batches of returned officers have just lately appeared and are again settling down to the pre-war appointments. We do not doubt that the military occupation of so important a district and the large number of Indian natives employed in developing the country call for a relatively large medical staff, but it should be remembered that the contract officers were engaged only for war work, and certainly from what one can gather a considerable number of regular R.A.M.C. men are still being given preference in returning home. The whole question is one which might and should be looked into, considering the length of time since armistice was signed and the number of young special reserve officers now available.

### THE 'TIMES' ATLAS.

WE would draw attention to the issue of the first batch of maps of the *Times* Survey Atlas of the world, which will at once bring to those inspecting them fresh evidence of the thoroughness and excellence with which our contemporary carries out any task to which it may set its hand. The introduction throughout the work, for the first time in a world atlas, of "orographical" colouring is undoubtedly a valuable step. Colour is a medium of expression far too valuable to be worked, as it usually has been, in unscientific adornment. Dr. Bartholomew, Cartographer to the King, who has been responsible for the preparation of this work, has used colour solely for the representation of heights and depths, with the result that a striking picture of every land is presented. The adoption of the "Loose-leaf" system, which enables the atlas to be bound with ease at home, is also a development holding a great general appeal.

### THE TASMANIAN MUDDLE.

IT will be remembered that as a result of the British Medical Association calling out its members who were honorary medical officers to the Hobart Hospital, after the controversy as to the alleged financial unsuitability of some of the patients, the Government succeeded in staffing the hospitals with five salaried medical men, and appointed a Mr. V. R. Rattan as Surgeon-Superintendent. The Association raised a doubt as to the correctness of Mr. Rattan's credentials, and a Royal Commission was appointed to investigate them, which pronounced in favour of the authenticity of an American diploma issued by the Harvey Medical College in 1907. Although the Commission was presided over by a judge, the evidence upon which the decision was given did not appear to everybody to be very convincing. On the most favourable showing the Harvey Medical College could not claim to be a medical educational authority of any great standing, and, in fact, only existed five years. The diploma granted to Mr. Rattan was dated only two months after the College was given a charter, and possibly applied to medical attainments acquired elsewhere. The British Medical Association has since been quite busy looking into the records of ephemeral medical colleges and the personal history of their officers. It is now alleged that one of the officers who signed Mr. Rattan's diploma in 1907 has been locked up for selling bogus medical qualifications in Chicago. There seem to be some grounds for further investigation of the charges brought by the Association.

### DOCTOR AND DOG FANCIER.

DR. J. SYDNEY TURNER, who died at his residence at Anerley on the 16th inst. at the age of 76 years, was well known in the medical profession, and had served as President of the south-eastern branch of the British Medical Association and of the Sydenham District Medical Society. He was a Guy's man and was at 21 a house surgeon at his hospital. But Dr. Turner was by far better known in the world of dogs, and was Chairman of the general committee of one of the most autocratic bodies in this country—the Kennel Club. During his long tenure of that office many a person implicated in some shady transaction connected with doggy affairs must have come before him in great trepidation, for he who errs in this particular circle, and is found out, is quickly cast into outer darkness and the dog-shows know him no more. Dr. Turner was a great dog-lover, and at one time or other went in for all breeds except toy dogs. Since 1863 the doctor has exhibited bull and black-and-tan terriers, beagles, fox-terriers, St. Bernards, retrievers, Yorkshire terriers, mastiffs, Blenheim spaniels, Esquimaux and bloodhounds. Dr. Turner had a long and honourable career, and he will be greatly and widely missed.

### SPRAYING INFLUENZA CONTACTS.

According to a report of the Bombay Medical Officer of Health, a steam spraying-chamber has been erected for the purpose of dealing with contacts of influenza. The main principle is that, by means of an aspirating device, a suitable disinfectant contained in bottles is sprayed into the chamber by steam jets, to which bottles are connected. The chamber is of a size which is suitable for treating ten persons at one time. The steam is obtained from a boiler working at 50 lb. pressure, and arrangements have been planned so that an existing boiler with an Equifex disinfector has been made use of. The disinfectant used is a solution containing Lysol .5 per cent., normal saline solution .8 per cent., and zinc sulphate solution 1 per cent. The fluid jets are arranged so as to allow one litre of the solution to be delivered in 15 minutes, which is the maximum duration of the sitting for the persons being treated, and instructions are given that the spray is to be inhaled vigorously. Inhalation once a day for three consecutive days is the system now being done, but it is difficult to force contacts to attend more than once, chiefly because the value of the inhalation as a preventive measure is not appreciated. Care is taken to ensure thorough ventilation of the chamber after each sitting, and also prevent the rooms being hot or stuffy during the sitting. A medical officer is in charge of the operations, and is present while the treatment is being carried out, but the district registrars in charge of municipal dispensaries in Bombay discover contacts and mild cases and persuade them to take this treatment. Not only contacts of influenza and mild cases of influenza, but contacts of other diseases, such as measles, moles, and cerebro-spinal fever can be dealt with in such chambers if occasion arises.

### SLEEPING SICKNESS.

A notice of what is described as a reassuring statement by the Ministry of Public Health, at Rowe, deals with the number of cases of sleeping sickness in Italy. It says that reports have much exaggerated the prevalence of this disease, and that there are at present only twenty known cases in the country, and that the majority of these cases have now recovered. A report so greatly abbreviated as is this one is of little value save to provoke one's curiosity; were these cases imported from Africa, in which event there is no cause for alarm, or are any of them indigenous, when the occurrence of even one case, not to speak of twenty, is a matter of the very gravest moment. It is also reported that certain cases are under observation in a London hospital, where the symptoms suggest meningitis (*sic*), the one mentioned being sleepiness, and here too a note is added that examination of these patients' blood fails to show the presence of a trypanosome. Doubtless this is our old friend botulism, now known to be encephalitis lethargica, who is once more being forced upon an only too willing public, to make our flesh creep.

### SOME WAGES.

Hospital authorities in this country have had some recent experience of the increasing cost of maintaining an institution, especially in the matter of salaries and wages. But our rates of remuneration, high as some may think them, are considerably lower than in Australia. The Prince Alfred Hospital, Sydney, has entered into a new agreement with the Hospital and Asylum Employees' Union, which for the most part is very liberal towards the hospital employee. It has, however, the curious result of placing the unskilled worker generally on an equality with the skilled worker, and sometimes in a superior position. The agreement generally is generous as regards hours of work, holidays, and payment during sick leave. The schedule of rates of remuneration covers about ninety specified posts, not including doctors, nurses, massage workers, and the secretary or his assistant. A storekeeper will get £4, rising to £5 a week; a book-keeper the same; female shorthand writers and typists, £2 10s; first instrument attendant, £4 15s. to £5 10s.; head hall porter, £4 2s. 6d.; chief dispenser, £6 to £6 5s.; other dispensers, £3 10s. to £4 7s. 6d.; housekeeper, £2 7s. 6d., with board and lodging; junior male clerks (under 21), £2 7s. 6d. to £2 17s. 6d.; the same (over 21), £3 7s. 6d. to £3 12s. 6d.; junior female clerks (under 21), £1 7s. 6d. to £2 5s.; the same (over 21), £2 10s. In Sydney it is seemingly an advantage to be male and unskilled. Where calculations involve the question of board and lodging, this item is valued at 17s. 6d. a week.

### THE CAMPAIGN AGAINST CONSUMPTION.

Reporting on the recent conference of the National Association for the Prevention of Tuberculosis, the Sheffield Corporation delegates, in reviewing their impression of the conference, declare that it was gratifying to find that in France and in the United States, as well as in this country, organised efforts are being made to stamp out tuberculosis, and there is a free and frank interchange of knowledge gained in these countries by those efforts. There is still some difference of opinion among doctors as to the predisposing causes of tuberculosis, but, generally speaking, methods of prevention and treatment are becoming standardised as the scientific study of the disease develops. A strong effort is being made to enlist in the campaign against tuberculosis the services of V.A.D. nurses who, having gained experience during the war, and now being demobilised, are at liberty to take up duties in relation to tuberculosis. If this proposal is pressed, the Corporation delegates think great care should be exercised in fitting in their work with that of the local health authority.

### A FUND COMMEMORATED.

The French custom of bestowing decorations on cities which either suffered or were the scenes of heroic defence in the war is not usual in this country. But a somewhat similar event has occurred in connection with the Royal Victoria and West Hants Hospital, where a tablet has been unveiled to commemorate the fact that the Bournemouth Hospital Saturday Fund has raised £20,000 since its establishment for this hospital. On the whole, we think, the architectural commemoration of individuals has more in its favour, since they are thus honoured when their work is completed. The Bournemouth Hospital Saturday Fund, however, is still active and expects to grow. Would not a better memorial have been to associate a bed, or even a ward, with its name, since the bed or the ward, like the fund itself, remains a living force in the life of the hospital?

### THE SHEFFIELD HOSPITAL COUNCIL.

While much has been heard of hospital coordination, there have been few data hitherto by which to judge the effectiveness of the joint councils that have been formed. We therefore await with interest the expected report of the committees established by the Sheffield Joint Consultative and Advisory Hospital Council, which has been in existence for six months. The problems of waiting-lists, of finance, have been considered, and we now want to hear the proposals for relieving them.

### PRESENTATION TO MR. W. C. YELL.

The members of the board of the East Suffolk and Ipswich Hospital have presented a piece of plate to Mr. W. C. Yell for his work for the wounded during the war. Mr. J. D. Cobbold, the chairman, in making the presentation, described Mr. Yell's regular visits to the men in the wards and the excursions which he provided for the convalescents. Mr. Yell has had experience of hospitals in different parts of the world, including the Antipodes, and is now trying to raise £100, to be divided equally between the memorial fund and the general work of the hospital.

### RED CROSS BEDS IN LOCAL HOSPITALS.

The Governors of the Royal Surrey County Hospital have agreed to the proposal of the Surrey branch of the Red Cross Society to provide treatment for discharged soldiers and their relations. Out of its income, therefore, the Surrey branch will maintain at least ten beds in the same way that an individual may name a bed. The number of beds to be maintained will be fixed each year, and also the sum necessary to maintain them will be fixed periodically. Supposing an extension to be made, the Surrey branch will adopt the new building for its war memorial. The wish of the branch that a course of training for V.A.D. nurses should be held at the hospital is also being complied with.

### PUBLICITY IN VILLAGES.

The difficulty in launching the proposed scheme for a cottage hospital for Hugglescote, Notts, illustrates a point which is too little recognised in rural districts. The proposal was put forward by responsible people, the profession supports it, and the colliers and local workers in the building trade have severally promised measures of support. But when a meeting was held to report progress and to further the scheme only a handful were present. The difficulty really is not want of co-operation, but want of publicity. It is so difficult to advertise any meeting in a small place that probably most of the residents were unaware of the occasion.

### THE STANDARDISATION OF SALARIES.

Lambeth Board of Guardians is endeavouring to secure the support of other London boards in the Metropolis for its proposal that a uniform salary should be paid to every officer of the same grade throughout the Metropolitan area of the Poor-law service. At the present time the different boards compete for the services of officers by offering higher salaries and, in consequence, the highest bidder secures plenty of applications. This disparity is particularly noticeable among medical officers and nurses. In some parishes staff nurses are paid at a higher rate than sisters in others, and there is little wonder that frequent changes in staffs occur at our Poor-law infirmaries. If the matter was dealt with by a conference of representatives of the different boards of guardians, it should not be difficult to frame an equitable scale of salaries which would apply to every grade of officer and thus give an incentive to these officials to remain in their posts, except for the prospect of promotion. Medical superintendents and matrons would welcome some such proposal, as their administration is handicapped by frequent changes in the medical and nursing staffs.

### THIS WEEK'S DRUG MARKET.

Business is fairly brisk and, generally speaking, the upward price tendency continues. Several articles which had been fairly stable in price for some time have moved upwards, as, for example, aspirin, salicylic acid and salicylate of soda. Camphor still hangs fire, and an arrival of synthetic camphor via Holland is significant. There is little change in the position of bromides, but larger quantities have arrived from Germany. Ergot of rye seems likely to be still dearer. Eucalyptus oil is still in good demand and the value is maintained. Lemon oil and bergamot oil are much dearer. Best quality olive oil is scarce and dearer. Potassium permanganate is scarcer and the price has advanced. At the recent public sale of drugs in Mincing Lane higher prices were paid for Cape aloes, cassia fistula, honey, ipecacuanha and sarsaparilla.

